# Peripheral trauma and risk of dystonia: What are the evidences and potential co-risk factors from a population insurance database?

**DOI:** 10.1371/journal.pone.0216772

**Published:** 2019-05-10

**Authors:** Antonella Macerollo, Mark J. Edwards, Hui-Chun Huang, Ming-Kuei Lu, Hsuan-Ju Chen, Chon-Haw Tsai, Jui Cheng Chen

**Affiliations:** 1 Department of Neurology, The Walton Centre NHS Foundation Trust, Liverpool, United Kingdom; 2 School of Psychology, Faculty of Health and Life Sciences, University of Liverpool, Liverpool, United Kingdom; 3 Department of Molecular and Clinical Sciences, St George’s University of London, London, United Kingdom; 4 Department of Neurology, China Medical University Hospital, Taichung City, Taiwan; 5 School of Medicine, China Medical University, Taichung City, Taiwan; 6 Management Office for Health Data, China Medical University Hospital, Taichung City, Taiwan; Medical University of Innsbruck, AUSTRIA

## Abstract

**Background:**

Dystonia is a neurological syndrome typically resulting in abnormal postures.

**Objectives:**

We tested the role of physical injury as potential risk factor for development of dystonia using The National Health Insurance Research Database of Taiwan.

**Methods:**

We identified 65704 people who were coded in the database as having had peripheral traumatic injuries (ICD-9-CM 807–848 and 860–959) in the year 2000. Patients with traumatic brain or spine injuries were excluded from analysis. We matched them using purposive sampling with 65704 people in the database who had not suffered peripheral trauma. We looked then at the incidence of dystonia occurring at least 1 year from the date of the peripheral trauma until 2011. Psychiatric symptoms (depression and anxiety) and sleeps difficulties have been investigated as potential covariates.

**Results:**

We found 189 patients with dystonia (0.28%) in the trauma group, and 52 patients with dystonia (0.08%) in the non-trauma group. Trauma was independently associated with dystonia (adjusted HR = 3.12, 95% CI = 2.30–4.24). The incidence density of dystonia in the trauma group was 2.27 per 10000 person-years, while it was 0.71 per 10000 person-years in the non-trauma group Beyond the peripheral trauma, other variables associated to the incidence of dystonia included female sex, aged 40 years and above, depression and sleep disorders.

**Conclusion:**

These data from a large population dataset support traumatic injury as a risk factor for the development of dystonia.

## Introduction

Dystonia is a movement disorder characterized by involuntary, sustained and patterned contractions of muscles typically resulting in abnormal postures [1].

Previous epidemiological studies have sought to determine environmental risk factors for the development of dystonia, in particular of isolated cranio-cervical dystonia.

The Italian Movement Disorders Study Group [2] performed a case control study of 202 dystonia patients and 202 controls matched for age and sex. They found physical trauma as an independent risk factor for the development of dystonia. However, a recent larger study by the authors of the Italian Dystonia Registry on 664 patients did not confirm this association [3]. It is essential to keep in mind that these two studies investigated different types of trauma. Indeed, the Italian Movement Disorders Study Group [2] divided the trauma into central with associated loss of consciousness and peripheral or extracranial injury. Of note, only the trauma associated with loss of consciousness was found to be significant with respect to dystonia [2]. The study performed by the authors of the Italian Dystonia Registry investigated extracranial injuries, which were divided into upper limb/lower limb/neck-trunk trauma [3]. This methodological difference might explain the different results. Defazio et al. discovered that 26% of cases had developed dystonia in the same site as the injury and 74% of cases exhibited dystonia in a separate site [3].

Newman et al. tested the role of central trauma with loss of consciousness in developing dystonia in a case control study of 184 patients and 1048 controls [4]. Head injury with loss of consciousness was found an independent risk factor for the development of cervical dystonia with the odds ratio of 4.8 (p = 0.004) in an analysis including matched controls and of up to 8.3 (p<0.001) in an analysis including a larger control group [4].

On the contrary, a multicenter case-control study performed by Martino et al. on 177 cranial dystonia patients and 217 age-matched controls with primary hemifacial spasm did not show any association between a prior head trauma and cranial dystonia [5]. Recently, a case control study of 67 cervical dystonia patients and 67 age-matched healthy siblings showed increased frequency of a history of car accidents with hospital attendance (OR 10.1, 95% CI 2.1 to 47.4,p = 0.004) and surgical episodes (OR 6.5, 95% CI 1.76 to 23.61, p = 0.005) in the cervical dystonia group [6].

Minor peripheral trauma has been previously studied in relation to a specific type of dystonia labelled ‘causalgia-dystonia’ [7, 8]. The latter is characterized by fixed postures [9] as well as unusual body distribution given the age at onset (e.g. leg dystonia in an adult) [10, 11]. Severe pain, similar to that present in chronic regional pain syndrome type 1 (CRPS1), is commonly associated to these dystonic postures [12]. The absence of task specificity as well as geste antagonist, the poor response to botulinum toxin [10] and the common involvement of the limbs [11] are typical clinical features of the “causalgia-dystonia” [7]. The pathophysiology is still under debate and there are some suggestions that it is a form of functional dystonia [7]. A neurobiologically focused model of the genesis of functional neurological disorders, including functional dystonia, has highlighted three key processes: abnormal self-directed attention (self-monitoring), abnormal beliefs/expectations and abnormal sense of agency [13].

Contrary, Frei et al. highlighted the organic nature of fixed dystonia in a group of 9 patients with posttraumatic cervical dystonia. These patients showed abnormal head postures within 7 days after cervical injury [14]

Overall, the pathophysiology of the fixed dystonia is still not clearly defined and several authors have tried to identify potential biomarkers that might help in reaching the correct diagnosis. In this regard, Macerollo et al. have demonstrated neurophysiological parameters as well as reaction time measures which might help to differentiate functional fixed dystonia from non-functional cases [15].

This study took advantage of a medical insurance database covering almost the entire population of Taiwan to study the cumulative incidence of dystonia in a group of patients with previous history of peripheral trauma. We expected to find an increased risk of dystonia in patients exposed to physical trauma. We were also interested in other factors that might covary with this risk. In particular, we investigated psychiatric disorders (depression and anxiety) and sleep disturbance since they are frequent comorbidities in patients affected by dystonia [7, 14, 15]. In regard to these comorbidities, Bermann et al. found that levels of depression, anxiety and social anxiety occurred in all groups of adult-onset focal dystonia phenotypes, although anxiety and social anxiety were higher in patients with cervical or laryngeal dystonia than in patients affected with upper cranial dystonia [16]. In line with these results, Zurowski et al confirmed the high incidence of anxiety disorders, especially social phobia and major depressive disorder in dystonia patients [17]. Additionally, these authors highlighted the presence of deficits in emotional processing in some dystonia populations [17]. Of note, Zurowski et al. found that the onset of psychiatric disturbances have an early onset in dystonia, even earlier than the motor dysfunction [17]. These findings support the hypothesis that the pathophysiology of dystonia itself might be involved in the genesis of psychiatric disturbances [17]. Sleep difficulties are another common comorbidity in primary dystonia as it has been showed by Avanzino et al. [18]Indeed, impaired quality of sleep were showed by higher scores of the PSQI (Pittsburgh Sleep Quality Index) in patients with cervical dystonia (CD) compared to the healthy controls, although it was partly confounded by the results of the Back Depression Inventory [18]. This confounded effect is another proof of the importance of depressive symptoms in CD.

## Methods

### Data source

The National Health Insurance (NHI) program is a universal insurance program established in 1995 in Taiwan, joining together 13 separate insurance-backed systems and providing comprehensive coverage for medical for care up to 99% of Taiwan’s population by the end of 2014. The National Health Insurance Research Database (NHIRD) managed by the Taiwanese National Health Research Institutes (NHRI) contains all the claim data from insured citizens, including beneficiaries’ demographics, dates of clinical visits, details of prescriptions as well as diagnosed diseases coded according to the International Classification of Diseases, Ninth Revision, Clinical Modification (ICD-9-CM). We made use of data from the NHIRD for the period of 2000–2011, which contains medical reimbursement claims from the NHI program for 1 million enrollees. Our study used anonymized data from the NHIRD and was exempted from full review by our institutional research ethics committee.

### Study population

A retrospective cohort study was conducted with two study groups: a trauma group and a non-trauma group. Patients with traumatic brain or spine injuries were excluded from analysis. The trauma group consisted of patients with a first-time diagnosis of trauma (ICD-9-CM 807–848 [fracture of trunk; fracture of upper limb; fracture of lower limb; dislocation; sprains and strains of joints and adjacent muscles] and 860–959 [Internal injury of chest, abdomen, and pelvis; Open wound of head, neck, and trunk; Open wound of upper limb; Open wound of lower limb; Injury to blood vessels; Late effects of injuries, poisonings, toxic effects, and other external causes; Superficial injury; Contusion with intact skin surface; Crushing injury; Effects of foreign body entering through orifice; Burns; Injury to nerves and spinal cord; Certain traumatic complications and unspecified injuries]). The index date for the patients in the trauma group was the date of their first diagnosis of trauma. We excluded patients with the following characteristics: missing information on age and/or sex, aged less than 20 years, a diagnosis of dystonia before the index date, fracture of skull or spine (ICD-9-CM 800–806) or intracranial injury (ICD-9-CM 850–854) diagnosis before endpoint date, less than one-year of follow-up from the date of the trauma. The non-trauma group was also identified from the insured people without any trauma diagnosis (ICD-9-CM 800–959) by propensity score matching methods [19].A logistic regression model was used to estimate the probability of assignment and to calculate the propensity score for each person, incorporating sex, age, depression, anxiety, sleep disorders and the diagnosis year in the model. For each patient with trauma, one person without trauma was selected from the nearest propensity score by greedy algorithm for the non-trauma group.

The primary outcome measure was the first occurrence of dystonia (ICD-9-CM 333.6, 333.7, and 333.8) during the follow-up period.

We excluded patients who developed dystonia within one year of the index date of trauma. The aim was excluding patients with a rapid onset of dystonia after injury, who might suggest a diagnosis of functional dystonia or dystonia secondary to brain injury. Participants were followed from the index date to the date of endpoint (i.e., until onset of dystonia, withdrawal from the insurance system or to December 2011).

Demographic factors included sex and age (in groups of 20–39, 40–59 and 60 years or older). We looked specifically for comorbidity with depression (ICD-9-CM 296.2, 296.3, 300.4, and 311), anxiety (ICD-9-CM 300.00) and sleep disorders (ICD-9-CM 307.4 and 780.5) present before the onset of dystonia [14].

### Statistical analysis

Data were expressed as frequencies and percentages for categorical data and as means and standard deviations (SD) calculated for continuous variables. We compared the distributions of sex, age and comorbidity between the trauma group and the non-trauma group. The standardized mean difference was calculated by the pooled value to determine whether the difference between the 2 groups was significant. A value of 0.1 or less was considered as a negligible difference between two groups [19]. The incidence density was calculated as the number of dystonia incident identified during the follow-up period divided by the total person-years for each group by sex, age and comorbidity. A Kaplan-Meier analysis was used to calculate the cumulative incidence rates of dystonia in trauma and non-trauma groups. The log-rank test was used to analyze the differences between the survival curves. The association between trauma and risk of dystonia was assessed using Cox proportional hazards regression adjusted for sex, age and comorbidities. We also evaluated the association of trauma and the risk of dystonia in various subgroups according to sex, age and comorbidities. Hazard ratios (HRs) and 95% confidence intervals (CIs) were calculated for quantifying the risk of dystonia after adjustment for sex, age and comorbidity. Analyses were performed using SAS (SAS, version 9.4; SAS Institute, Inc). The statistical significance level was 2-sided p-value<0.05.

## Results

We enrolled 65704 patients with trauma and 65704 without trauma in the year 2000. Characteristics of trauma group and non-trauma group are listed in Table 1. There were no significant differences in the distribution of sex, depression, anxiety and sleep disorders for the two groups. The mean age in the trauma group was 41.4 (SD = 15.3) years and 55.6% of patients were male.

**Table 1:** Baseline demographic factors and comorbidity of study participants according to trauma status.

	Trauma group N = 65704		Non-trauma group N = 65704		Standardized mean difference
Characteristics	n	%	n	%	
Sex					
Women	29202	44.4	29201	44.4	<0.001
Men	36502	55.6	36503	55.6	<0.001
Mean age (SD), year	41.4	(15.3)	41.7	(15.8)	0.017
Comorbidity					
Depression	472	0.72	472	0.72	<0.001
Anxiety	298	0.45	314	0.48	0.004
Sleep disorders	1349	2.05	1333	2.03	0.002

Abbreviation: SD, standard deviation

**Fig 1:**
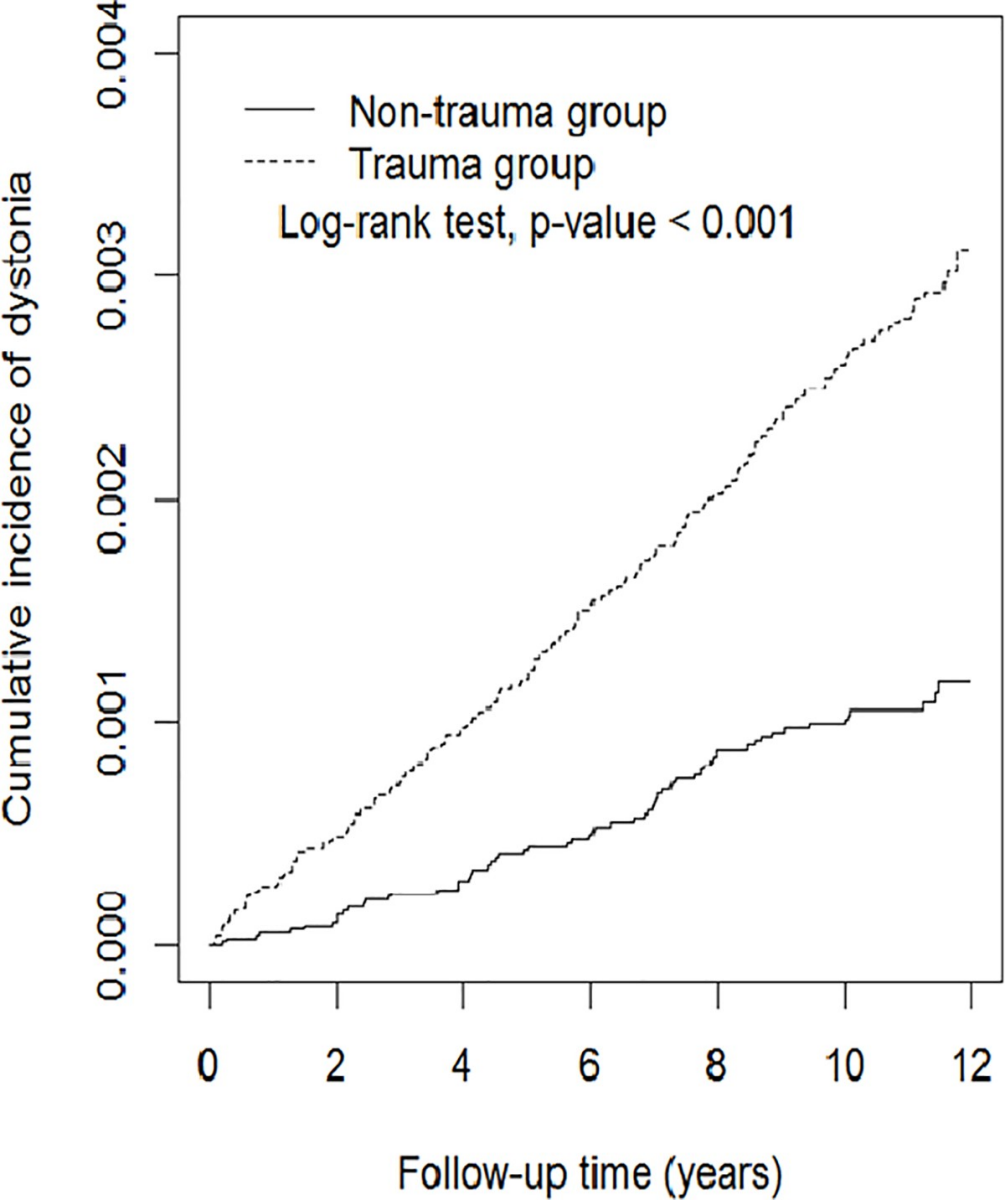
Cumulative incidence curves of dystonia for groups with and without trauma.

Cumulative incidence curves of dystonia according to trauma status are illustrated in Fig 1. We used a log-rank test to examine cumulative incidence of dystonia between groups with and without trauma. The cumulative incidence of dystonia was found significantly higher in the trauma group than in the non-trauma group (p<0.001).

During the study period, there were 189 patients with dystonia diagnosed (0.28%) in the trauma group and 52 patients with dystonia diagnosed (0.08%) in the non-trauma group. The incidence density of dystonia in the trauma group was 2.27 per 10000 person-years, while it was 0.71 per 10000 person-years in the non-trauma group (Table 2). We used a multivariate Cox proportional hazards regression analysis and found that trauma was independently associated with dystonia (adjusted HR = 3.12, 95% CI = 2.30–4.24). Female sex, aged 40 years and above, depression and sleep disorders were also independent risk factors for developing dystonia (adjusted HR = 1.52, 95% CI = 1.18–1.97; adjusted HR = 2.16, 95% CI = 1.65–2.81; adjusted HR = 3.58, 95% CI = 1.78–7.20; and adjusted HR = 2.43, 95% CI = 1.42–4.17, respectively).

**Table 2:** Cox model measured hazard ratios and 95% confidence interval of dystonia associated with trauma and covariates.

Characteristics	Event no.	Person-years	Incidence density^#^	HR (95% CI)	
				Univariate	Multivariate^†^
Trauma					
No	52	733710	0.71	ref	ref
Yes	189	831509	2.27	3.16 (2.33–4.30)	3.12 (2.30–4.24)
Sex					
Women	131	706526	1.85	ref	ref
Men	110	858693	1.28	1.44 (1.12–1.86)	1.52 (1.18–1.97)
Age, years					
20–39	90	887137	1.01	ref	ref
≥ 40	151	678082	2.23	2.22 (1.71–2.88)	2.16 (1.65–2.81)
Comorbidity					
Depression					
No	231	1555364	1.49	ref	ref
Yes	10	9855	10.2	6.95 (3.69–13.1)	3.58 (1.78–7.20)
Anxiety					
No	237	1558696	1.52	ref	ref
Yes	4	6523	6.13	4.09 (1.52–11.0)	1.57 (0.55–4.44)
Sleep disorders					
No	224	1537232	1.46	ref	ref
Yes	17	27987	6.07	4.25 (2.59–6.95)	2.43 (1.42–4.17)

^†^ Multivariate Cox proportional hazards regression model including trauma, age (categorical), depression, anxiety, and sleep disorders.

Abbreviation: HR, hazard ratio; CI, confidence interval.

^#^ per 10000 person-years

To further assess the robustness of our results, we stratified the study population based on sex, age group and comorbidity (Table 3). In the subgroup analysis, patients with trauma were consistently associated with a higher risk of dystonia in both sexes and all age groups compared with the study population without comorbidity.

**Table 3:** Incidence density and hazard ratios of dystonia according to trauma status stratified by sex, age, and comorbidity.

	Trauma group			Non-trauma group			Compared to the non-trauma group	
							HR (95% CI)	
Characteristics	Event no.	Person-years	Incidence density^#^	Event no.	Person-years	Incidence density^#^	Crude	Adjusted ^‡^
Sex								
Women	102	376140	2.71	29	330386	0.88	3.06 (2.03–4.62)	2.98 (1.97–4.51)
Men	87	455369	1.91	23	403324	0.57	3.29 (2.08–5.21)	3.25 (2.05–5.15)
Age, years								
20–39	70	469333	1.49	20	417803	0.48	3.07 (1.87–5.05)	3.07 (1.87–5.05)
≥ 40	119	362176	3.29	32	315907	1.01	3.19 (2.16–4.71)	3.15 (2.13–4.65)
Comorbidity status ^†^								
No	169	809811	2.09	45	716124	0.63	3.27 (2.35–4.54)	3.24 (2.33–4.51)
Yes	20	216981	9.22	7	17586	3.98	2.31 (0.98–5.48)	2.24 (0.95–5.32)

Abbreviation: HR, hazard ratio; CI, confidence interval.

# per 10000 person-years

† Patients with any one of depression, anxiety, and sleep disorders were classified as the comorbidity group.

‡ Model mutually adjusting for sex, age, depression, anxiety, and sleep disorders.

## Discussion

We have found that dystonia showed a higher incidence in patients with prior peripheral physical trauma. Consequentially, these results support the hypothesis that peripheral trauma might be a risk factor in developing dystonia. Our results are in line with previous studies. Data from cohorts acquired from specialist movement disorder clinics have suggested that physical trauma may be a risk factor for the development of isolated dystonia [2, 20, 21].

The case control study of 202 dystonic and 202 age and sex matched healthy controls performed by The Italian Movement Disorders Study Group [2] identified the physical trauma as an independent risk factor for the development of dystonia. In particular, an association between head or facial trauma with loss of consciousness and dystonia was found by these authors [2]. Further, there was a significant association between the local body injury and the body region affected by dystonic postures [2]. Italian Dystonia Registry Team did not replicate these results [3]. Indeed, the authors reported trauma in 8.7% of the dystonia patients. Moreover, 74% patients reported the trauma in a different location than that of the dystonia [3]. However, there were wide differences in methods including data analysis, type of study populations (i.e. a control group in the first study) and protocols of collecting data. Both studies relied on patients’ recall for their data. Further, the Italian Movement Disorders Study Group and the Italian Dystonia Registry Team were focused on different types of trauma. Indeed, the first study analyzed the association between dystonia and non-severe head or facial trauma with or without loss of consciousness [2]. Trauma was defined as requiring medical assistance but not surgery [2]. Whereas, the study on the data of the Italian Dystonia R nfrregistry did not give any definition of trauma [2,3]. Similarly, both studies found eye disorders/symptoms associated with blepharospasm [2,3].

Multiple environmental events have been suggested to play a role in the development of different phenotypes of primary focal dystonia, including head, neck and limb trauma, upper respiratory tract infection, early childhood infection, eye disease, scoliosis and repetitive motor actions [22–28].

Our study used a large insurance database to assess the risk of development of dystonia in people experiencing physical trauma. We found physical trauma to be a risk factor for the development of dystonia in addition to other factors (age, female sex, depression and sleep disorders).

We attempted to select people who experienced trauma without brain or spinal cord injury.

We hypothesized that the majority of our patients had cranio-cervical dystonia based upon the high prevalence of this type of dystonia in the literature and in view of the age at onset. In particular, this assumption is supported by the predominance of females in patients with dystonia, in keeping with the known female predominance in people with isolated cranio-cervical dystonia and that dystonia risk increased with increasing age [29].

Trauma was an independent risk factor for the later development of dystonia. The risk of developing dystonia in patients with previous trauma was 2.59 per 10000 person-years compared to 0.97 per 10000 person-years in the comparison group. Furthermore, trauma was independently associated with dystonia (adjusted HR = 2.51, 95% CI = 1.89–3.34) after adjusted with comorbidities. This result suggests that the trauma itself is correlate to the development of dystonia and it is not influenced by other comorbidities. This hazard ratio is in line with that reported in previous cohorts of adult-onset isolated dystonia, confirming trauma as an independent risk factor for the development of dystonia [2, 4, 20]. In this regard, Defazio et al. [2] in their multivariate analysis on 202 cases and 202 age and sex matched control outpatients found that that head or facial trauma with loss of consciousness independently increased the risk of developing adult onset dystonia (OR = 3.5, 95% CI = 0.7 to 18, p = 0.13). Of note, family history of dystonia (95% Cl = 2.86 to infinite; ÷χ with continuity correction = 8.1, p = 0.004), and family history of postural tremor (OR = 7.45; 95% Cl 1.67 to 68.1; p = 0.003).) were also other independent risk factors for the development of dystonic posture in this cohort [2].

Newman et al. found that head injuries with loss of consciousness was an independent risk factor for the development of idiopathic dystonia (OR = 4.781; 95% CI = 1.626–14.060; p = 0.004) and the result was confirmed in a repeated analysis with a larger control group (OR = 8.286; 95% CI = 3.871–17.736; p = <0.001) [4].

Interestingly, Molloy et al. investigated different types of traumas including car accident with hospital admission and surgical procedures as potential risk factors for cervical dystonia [20]. These types of trauma were found more frequently present in cervical dystonia patients compared to unaffected siblings. Following multivariate analysis, car accidents with hospital attendance (OR = 7.3, 95% CI = 1.4 to 37.6, p = 0.017) and all surgical episodes (OR = 4.9, 95% CI = 1.24 to 19.31, p = 0.023) remained significantly associated with dystonia.

Depression, anxiety and sleep disorders have been found significantly associated with the later development of dystonia in our cohort as it was identified in previous studies [30, 31].

The strengths of our study were its population-based design, generalizability of findings and use of population-based data. Further, NHIRD records used a large sample size and had low loss to follow-up in the longitudinal design, including study and control cohorts. In addition, NHIRD covers a highly representative sample of Taiwan's general population because the reimbursement policy is universal and operated by a single-buyer, the government in Taiwan.

We recognized some limitations of our study. Firstly, the lack of possibility to ascertain the type of dystonia from the available data. There may be other factors that influence the development of dystonia that we did not record. We are aware that the lack of ability to associate the type of trauma to the type of dystonia is a significant limitation as it has been suggested that trauma in the affected area is thought to predispose to dystonia in that area.^2^ Additional limitations include the lack of knowledge of who determined the diagnosis of dystonia–movement disorders specialist vs neurologist vs primary care physician and how the patients were diagnosed with dystonia.

Further work is necessary to explore the mechanism of this potential association in experimental studies as well as further large-scale population studies to better understand the level of risk and interaction with other risk factors.
